# Percutaneous Dorsal Instrumentation of Vertebral Burst Fractures: Value of Additional Percutaneous Intravertebral Reposition—Cadaver Study

**DOI:** 10.1155/2015/434873

**Published:** 2015-06-02

**Authors:** Antonio Krüger, Maya Schmuck, David C. Noriega, Steffen Ruchholtz, Gamal Baroud, Ludwig Oberkircher

**Affiliations:** ^1^Department of Trauma, Hand and Reconstructive Surgery, Philipps University, 35043 Marburg, Germany; ^2^Spine-Unit, University Hospital Valladolid, 47008 Valladolid, Spain; ^3^Laboratory of Biomechanics, Department of Mechanical Engineering, University of Sherbrooke, Sherbrooke, QC, Canada J1K 2R1

## Abstract

*Purpose*. The treatment of vertebral burst fractures is still controversial. The aim of the study is to evaluate the purpose of additional percutaneous intravertebral reduction when combined with dorsal instrumentation. *Methods*. In this biomechanical cadaver study twenty-eight spine segments (T11-L3) were used (male donors, mean age 64.9 ± 6.5 years). Burst fractures of L1 were generated using a standardised protocol. After fracture all spines were allocated to four similar groups and randomised according to surgical techniques (posterior instrumentation; posterior instrumentation + intravertebral reduction device + cement augmentation; posterior instrumentation + intravertebral reduction device without cement; and intravertebral reduction device + cement augmentation). After treatment, 100000 cycles (100–600 N, 3 Hz) were applied using a servohydraulic loading frame. *Results*. Overall anatomical restoration was better in all groups where the intravertebral reduction device was used (*p* < 0.05). In particular, it was possible to restore central endplates (*p* > 0.05). All techniques decreased narrowing of the spinal canal. After loading, clearance could be maintained in all groups fitted with the intravertebral reduction device. Narrowing increased in the group treated with dorsal instrumentation. *Conclusions*. For height and anatomical restoration, the combination of an intravertebral reduction device with dorsal instrumentation showed significantly better results than sole dorsal instrumentation.

## 1. Introduction

The treatment of vertebral burst fractures without neurologic compromise is still controversial. Recent meta-analysis of the literature revealed that surgical treatment offered no advantage over nonsurgical one [1, 2]. Open posterior instrumentation is the most frequently used technique in the treatment of unstable traumatic thoracic and lumbar fractures. There is a general tendency towards minimallyu invasive approaches in various surgical disciplines [3]. Further, there is a wide consensus that the main treatment goal in young patients is anatomically correct reduction and solid fixation to maintain the restored sagittal balance. In the case of insufficient reduction, an open approach sometimes in combination with a 360° fusion is recommended. We know from several studies that complications in 360° fusion can be considerable and that there is a significant height loss at follow-up after cases of sole posterior instrumentation. Using minimally invasive surgery combining percutaneous intravertebral reposition and percutaneous instrumentation could be a solution for anatomically correct fracture reduction to avoid recompression or height loss.

Most studies focus on restoration of the sagittal balance. This is measured using the Cobb angle, mostly as a bisegmental angle. Repositioning of the endplate and the posterior fragments is underrepresented in most evaluations. The rationale behind surgical treatment should focus on the anatomical restoration of the vertebra. Verlaan et al. [4] stress the importance of endplate reduction using the so-called BAER-technique (Balloon Assisted Endplate Reduction).

The aim of the present study is to evaluate the additional value of repositioning and height maintenance of different combinations of dorsal percutaneous techniques in the treatment of traumatic burst fractures. Height restoration and maintenance after cyclic loading and the standard procedure of a dorsal instrumentation are compared to another accepted technique, namely, the combination of dorsal instrumentation with intravertebral repositioning using the SpineJack and cement augmentation. Two other groups are also evaluated: SpineJack stand-alone with cement augmentation and the combination of percutaneous dorsal instrumentation with SpineJack but without cement.

## 2. Materials and Methods

### 2.1. Specimens

To compare the different techniques it was necessary to create similar conditions in all groups. For this reason all devices were investigated on the same level of the spine using fresh frozen human cadaveric spines (T11-L3). Twenty-eight spine segments were used in total. Cadavers were ordered by anatomic gifts registry based on anonymized patients profiles. To reduce the risk of osteoporosis or minor bone quality we selected only male donors (mean age 64.9 ± 6.5 years) with no history of tumours, osteoporosis, arthritis, or medications that could lead to secondary osteoporosis. Specimens were stored at −20°C. Prior to surgery, CT scans were performed to identify any pathologies. The spines were dissected into segments (T11-L3) and the dorsal autochthonous musculature was removed. Care was taken to keep the interspinous and supraspinous ligaments intact. The tissues around the upper third of T11 and the lower third of L3 were completely removed to allow a stable embedding in Technovit 3040 (cold-curing resin for surface testing and impressions, Kulzer, Germany). For fracture generation, surgery and loading specimens were thawed in a 37° Celsius water bath and immediately frozen after intervention.

### 2.2. Fracture Generation

**Figure 1:**
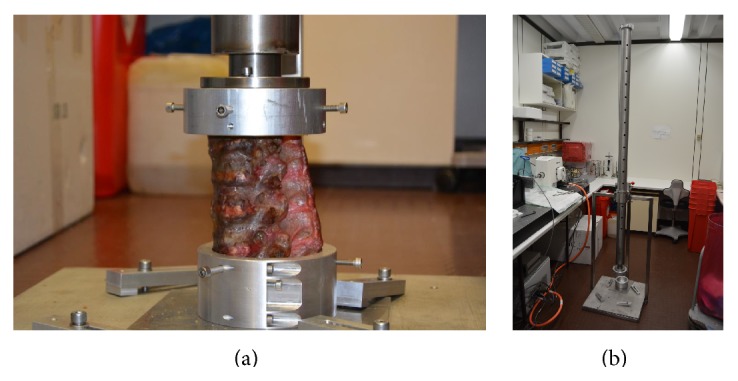
(a) Fixation of the embedded spine segments in the drop tower. A weight of 7 kg was dropped from a height of 1,7 meters. The displacement of the impactor was mechanically limited to 2.5 cm. (b) Complete drop tower.

In contrast to osteoporotic fracture models where axial loads are slowly increased to create fractures, a traumatic sudden impact had to be standardised to create comparable fractures. We decided to modify the technique described by Kallemeier et al. [5]. A drop tower was built allowing us to drop a load of 7 kg from a height of 1.7 meters on a horizontal aligned impactor (Figures 1(a) and 1(b)). The displacement of the impactor was mechanically limited to 2.5 cm. T11 and L3 were used to embed the segments, and the instrumentation ranged from T12 to L2. To assure that only L1 was broken, stress risers were generated by cutting the superior endplate and lamina of the first lumbar vertebra prior to impaction.

### 2.3. Restoration Measurements

All measurements were taken on CT scans to eliminate inaccurate measurements due to projection errors. CT scans were performed using 64-slice dual source scanner (Siemens Somatom Definition, Siemens, Forchheim, Germany). Multiplanar reconstructions were calculated and pictures were transferred into the clinical PACS server. Measurements were performed using IMPAX EE R20, AGFA Healthcare, Mortsel, Belgium. Vertebral heights were measured at the anterior and posterior walls as well as in the centre of the vertebral bodies in the midsagittal plane of the vertebral body according to the six-point method. Measurements were performed by an experienced surgeon and coauthor Ludwig Oberkircher according to clinical practice. Vertebral heights were measured before and after fracture as well as after treatment and cyclic loading. The bisegmental Cobb angle (angle between upper endplate T12 and lower endplate L2) was measured according to clinical studies.

The spinal canal compromise in the midsagittal plane was measured as a percentage of the minimum diameter before fracture (= 100%), after fracture, after surgical procedure, and after cyclic loading.

### 2.4. Experimental Groups

Four comparable groups were built according to three matching criteria.

- The main criterion for instability is fracture morphology. All fractures were classified according to the AO/OTA classification [6]. There were 16 A 3.1, 8 A 3.2, and 4 A 3.3 fractures. 4 A 3.1, 2 A 3.2, and 1 A 3.3 fractures were present in every one of the four groups.
- The second criterion is the grade of central deformation.
- The third criterion is size of the vertebral body to achieve comparable screw and rod sizes.

**Figure 2:**
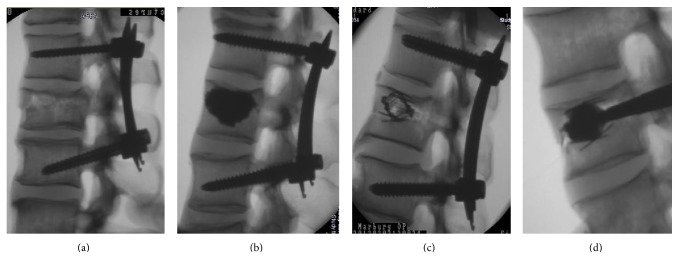
Experimental groups from left to right. (a) Posterior instrumentation with a percutaneous system (PI); (b) posterior instrumentation + SpineJack + cement augmentation (PI + SJ + CE); (c) posterior instrumentation + SpineJack without cement (PI + SJ); (d) SpineJack (Vexim, Balma, France) + cement augmentation (SJ + CE).

These four groups were treated in four different ways selected at random (Figure 2) as follows:

- posterior instrumentation (PI) with a percutaneous system (CD HORIZON Sextant II, Medtronic, Sofamor Danek),
- posterior instrumentation with a percutaneous system (CD HORIZON Sextant II, Medtronic, Sofamor Danek) + SpineJack (Vexim, Balma, France) + cement augmentation (PI + SJ + CE),
- SpineJack (Vexim, Balma, France) + cement augmentation (SJ + CE),
- posterior instrumentation with a percutaneous system (CD HORIZON Sextant II, Medtronic, Sofamor Danek) + SpineJack (Vexim, Balma, France) without cement (PI + SJ).

## 3. Surgical Technique

### 3.1. SpineJack (Vexim, Balma, France)

This implant concept is based on the “in situ fracture reduction” principle whereby an intravertebral body implant is expanded in situ to potentially restore the anatomy of the vertebral body mechanically. Afterwards, conventional polymethylmethacrylate (PMMA) bone cement is injected to stabilise the restored vertebra. The implant is made of titanium alloy. For the purpose of this study, the 5 mm device was used.

The device is inserted transpedicularly into the fractured vertebral body in an unexpanded format. After insertion into the VB, the implant is expanded using a specially designed tool. Longitudinal compression of the device causes the implant to open in the inferior-superior direction. All procedures were performed by the same surgeon (Antonio Krüger).

The length of the screws was measured by placing trajectories through the pedicles on the CT scans. Additionally the diameter of the pedicles was measured. The following screw lengths and diameters were used for instrumentation (5.5/50 mm; 6.5/50 mm; 6.5/55 mm; 7.5/60 mm). The longest and biggest screw possible was implanted. The following screw sizes were used in the different groups:

 PI: T12: 4 × 5,5/50; 2 × 6,5/50; 8 × 6,5/55,   L2: 4 × 5,5/50; 8 × 6,5/55; 2 × 7,5/60,
 PI + SJ + CE: T12: 6 × 6,5/50; 6 × 6,5/55; 2 × 7,5/60,   L2: 6 × 6,5/50; 4 × 6,5/55; 4 × 7,5/60,
 PI + SJ: T12: 4 × 5,5/50; 10 × 6,5/55 L2: 2 × 5,5/50; 4 × 6,5/50; 2 × 6,5/55; 6 × 7,5/60.

In all groups the spine segments were positioned prone without any traction or hyperextension. The spine segments were positioned prone for surgery but no attempt at external reposition was made. With the dorsal instrumentation used no instrumental reduction is possible. First, transpedicular guide wires were positioned using a C-arm that was rotated by 90° to visualise the spine in two planes. After the K-wires were positioned correctly, screws were placed in a Seldinger technique and rods were fixed with momentum screws. With combined procedures the pedicel screws were placed first. The SJ device was expanded after screw positioning and before fixation of the rods in the combined groups.

The same amount of cement was used to augment the vertebral bodies in all cases. A total of 5.4 mL (3 bone fillers) PMMA-Bone Cement (Cohesion Vexim, Balma, France) was used on each side. In a previous biomechanical study from our group height restoration was dependent on the cement volume used. This amount was used to completely fill the vertebral body. Leakage has not been observed. To exaggerate differences in the groups between those with cement and those without cement high volumes have been used.

### 3.2. Cyclic Loading

**Figure 3:**
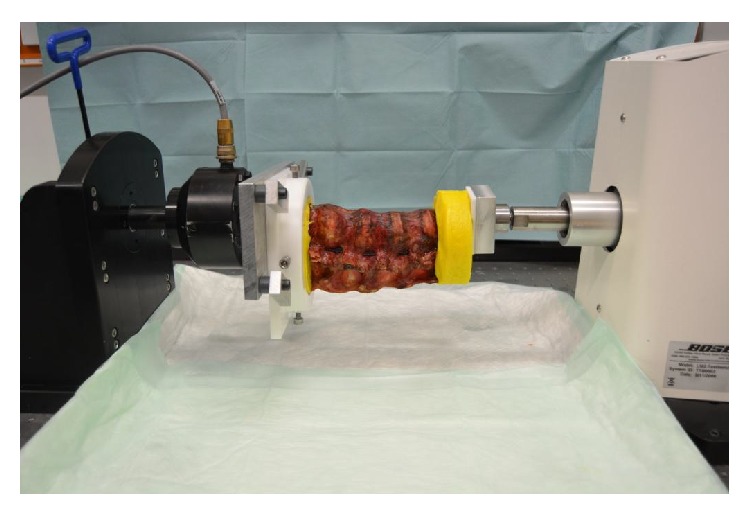
Positioning of the embedded spine segments in the servohydraulic loading frame.

After computed tomography cyclic loading was performed using a servohydraulic Test Bench (Bose Electroforce LM2 Test Bench) with 100000 cycles (3 Hz, 100–600 N) [7]. The spine segments were horizontally aligned (Figure 3). The endplates of T10 and L3 were embedded in technovit. The endplate of L3 was fixed in the loading machine. The load was applicate by a pivot that was centered midline on the anterior third of the vertebral body, allowing a bending moment.

### 3.3. Statistical Analysis

For all parameters determined, results are expressed as means, ranges, and ±SD. The test of significance between results from study pairs was conducted using Tukey's Test with significance *p* < 0.05. Tukey's Multiple Comparison Test is essentially a *t*-test, except that it corrects type I error rate when multiple comparisons are being made.

## 4. Results

Preparing and embedding spine segments worked favourably. Even after fracture, no repositioning or renewing of the fixation was required. All surgical procedures were carried out without complications or any instrument failure. No cement leakage was observed. In all cases, cyclic loading with 100000 cycles was performed, without technical imponderables. The CT scans taken before fracture showed no deformities in the L1 vertebrae.

### 4.1. Height Measurements

Values are expressed as percentages of measurements of the initial unfractured vertebral bodies (= 100%).  Table 1 gives an overview for all the data and measurements.

### 4.2. Measurements after Fracture

**Figure 4:**
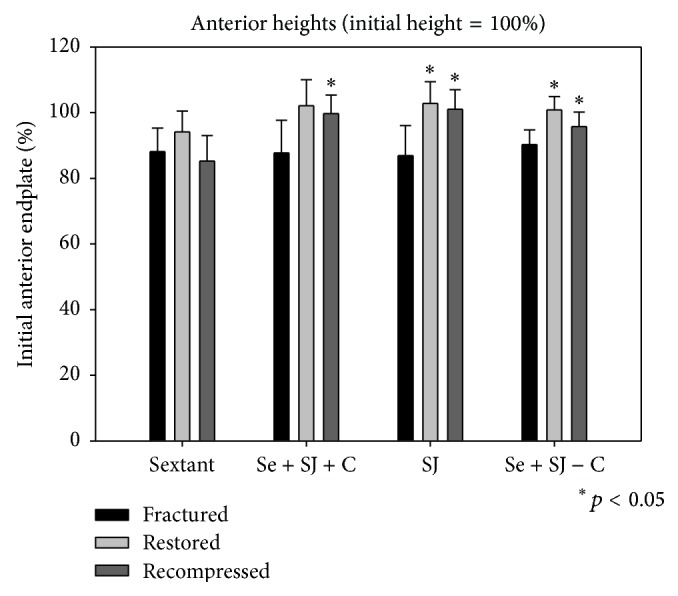
Bar graphs of the height measurements of the anterior wall of the vertebral body. The initial vertebral body height measured before fracture resembles 100%. Bars show the results for each of the four groups after fracture, after treatment, and after cyclic loading.

**Figure 5:**
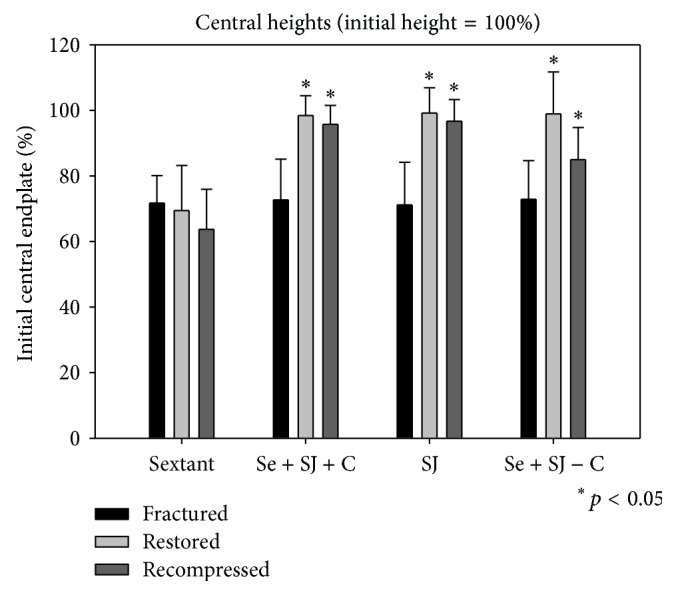
Bar graphs of the height measurements of the central midsagittal vertebral body (center of the endplates). The initial vertebral body height measured before fracture resembles 100%. Groups were matched according to central deformation. Bars show the results for each of the four groups after fracture, after treatment, and after cyclic loading.

**Figure 6:**
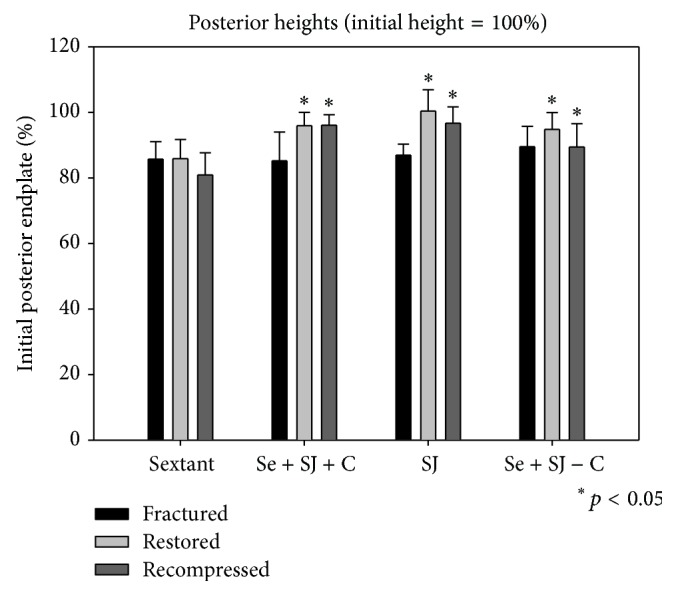
Bar graphs of the height measurements of the posterior wall of the vertebral body. The initial vertebral body height measured before fracture resembles 100%. Bars show the results for each of the four groups after fracture, after treatment, and after cyclic loading.

In all groups burst fractures with significant deformation were created. The central height loss was about 30% in all the groups (Figures 4–6).

For the average anterior, central, and posterior heights there were no significant differences between all groups (Tukey's Test (*p* > 0.05)).

### 4.3. Measurements after Surgical Procedure

The restoration of the vertebral anatomy was better in all the SJ groups. Most differences between the groups were significant (Figures 4–6).

For the central as well as the posterior heights there was a significant difference between the groups of PI versus PI + SJ + CE (*p* < 0.05), as well as for the groups of PI versus SJ + CE (*p* < 0.05) and PI versus PI + SJ (*p* < 0.05).

### 4.4. Measurements after Cyclic Loading

The measurements of the heights after cyclic loading show also that the vertebral anatomy was better in all the SJ groups (Figures 4–6). The restored anatomy was maintained. For the anterior and central as well as the posterior heights there was a significant difference between the groups of PI versus PI + SJ + CE (*p* < 0.05), as well as for the groups of PI versus SJ + CE (*p* < 0.05) and PI versus PI + SJ (*p* < 0.05).

### 4.5. Measurement of the Bisegmental Cobb Angle (Figure 7)

For the measurements of the bisegmental Cobb angle measured between the upper endplate of T12 and the lower endplate of L2 there were no significant differences between the groups (Tukey's Test (*p* > 0.05)) at all time points. The standard deviation was very high.

### 4.6. Measurement of the Narrowing of the Spinal Canal (Figure 8)

For the measurements of the average narrowing of the midsagittal spinal canal after fracture there were no significant differences between the groups (Tukey's Test (*p* > 0.05)). Nevertheless, the narrowing of the spinal canal increased in the posterior instrumentation group. In all the SJ groups the narrowing decreased but the difference was statistically not significant.

## 5. Discussion

Incomplete cranial burst fractures (AO classification type A 3.1.1) represent the most common fractures of the thoracolumbar spine in young patients [8]. The treatment of burst fractures without neurologic deficit remains controversial. Several reviews have shown that operative treatment is not superior to surgical treatment [1, 2]. Some clinical studies suggest short posterior instrumentation of these fractures [9]. Despite the lack of good evidence for surgical management of these fractures, the majority are treated surgically. Surgical treatment options include posterior, anterior, or combined posterior-anterior (360°) stabilisation [10]. Posterior stabilisation is widely used, technically easy, and associated with lower access morbidity [11]. Isolated posterior stabilisation may lead to loosening of the instrumented construct or loss of the height restoration achieved, since the anterior column contributes 80% of the stability of the spine [12–14]. Minimally invasive spine surgery with percutaneous positioning of the implants has additionally reduced the intraoperative risks [3]. These benefits have to be weighed against the limited advantages of intraoperative reposition besides patient positioning and traction.

Additional anterior stabilisation increases perioperative morbidity [15, 16]. At present, the appropriate method of stabilisation still remains unclear.

A systematic literature review shows [17, 18] that the choice of treatment is predominantly based on surgeons' individual preferences or those of an institution even if the level of evidence is low.

In a prospective randomised study, Korovessis et al. [16] compared (RCT) patients with burst fractures (L2–L4) treated by posterior stabilisation alone or combined posterior-anterior stabilisation. Clinical results were better in the posterior alone group. Remarkably, Korovessis et al. [16] did not recommend the use of posterior alone stabilisation because this stabilisation technique was not able to maintain the restoration of sagittal alignment.

One of the largest observational studies, by Reinhold et al. [8], classified 373 patients with type A 3.1.1 fracture according to AO classification without neurological deficit. 179 of these patients were operated on posteriorly only; 117 patients were treated with combined posterior-anterior procedure; 45 were treated with isolated anterior procedure, and 32 were treated conservatively. At follow-up, the posterior-only patients had a better functional and subjective outcome than the combined group. The overall complication rate was higher for the combined group (15.1%) compared to the posterior-only group (10%).

One of the greatest challenges is that clinical outcome is hard to correlate with sagittal restoration or radiologic outcome. The potential benefits of an improved sagittal alignment achieved by additional anterior stabilisation have to be weighed against the overall increased complication rate. In other words, is the rational or intellectual benefit worth the risks?

Using an intravertebral reduction device that can be placed transpedicularly reduces the risks of an anterior approach. This technique addresses the problem of it not being possible to treat the anterior column of the spine by dorsal instrumentation. In every instance in our study in which the SJ was used it showed its capabilities as a reduction device. Compared to one of the gold standards in the treatment of burst fractures, our results showed that there is an additional value in using SJ combined with dorsal instrumentation. The main aim of our study was to compare sole dorsal instrumentation with the combination of dorsal instrumentation with SJ. The combination of balloon kyphoplasty with dorsal instrumentation is an accepted procedure [19]. Using a percutaneous technique in burst fractures is a current method. Shorter operation times, reduced transfusion rates, and preserving the soft tissues are the main benefits [20]. Opponents of this technique criticise the limited feasibility for intraoperative mechanical reposition and the use of polyaxial screws. The use of monoaxial screws, posterior instrumental reposition, and a multilevel instrumentation (two above and two below) might have influenced the results of the posterior instrumentation group. We decided on using the least invasive dorsal instrumentation possible, while combining the advantages of both procedures.

To date the manufacturer's recommendation is that the vertebral body should be stabilised after reduction using cement augmentation. In our study the recommended PMMA cement was used. Opponents of PMMA argue that this cement should not be used in younger patients and that other more bioactive cements should be used. The use of calcium phosphate cements might be purely idealistic because clinical results are not encouraging [21]. Another argument against cement augmentation is that it could hinder the healing of the bone. The authors agree that the smallest amount of cement necessary for stabilisation should be used, but what the ideal cement volume is remains unclear. One potential advantage of the SJ may be that, combined with a dorsal instrumentation, it is stable enough to maintain height even without using supplementary augmentation. In our model high volumes of cement have been used. In addition to the pain control cement has a stabilizing factor in the treatment of burst fractures. The average amount used in a clinical study on osteoporotic burst fracture treated by balloon kyphoplasty was 8,4 mL [22]. The use of SJ might lead to a reduction of cement volumes. Clinical studies are necessary to underline this hypothesis.

The comparison of the dorsal instrumentation with the stand-alone augmented SJ showed that the height restoration and maintained height after cyclic loading were better in the SJ group. When placed in the context of clinical practice, this brings the importance of dorsal instrumentation in the treatment of compression fractures of the anterior column into question. Anterior pathology might be addressed better using a direct reduction device.

When measuring radiologic outcome, the anatomical restoration of the shape and endplates should be the goal. The bisegmental Cobb angle used to compare the clinical results might be of value in wedge compression fractures but in burst fractures it is of minor importance. The results of our measurements with a very large standard deviation were not significant and place doubt on the use of this measurement technique in laboratory practice. Specimens might change position in the time between load application and CT scan (Flexion/Extension; Lat. Bending-material relaxation). A missing loading apparatus for the CT scan and nonstandardized positioning can be an explanation for the high standard deviation of the Cobb angle.

**Figure 8:**
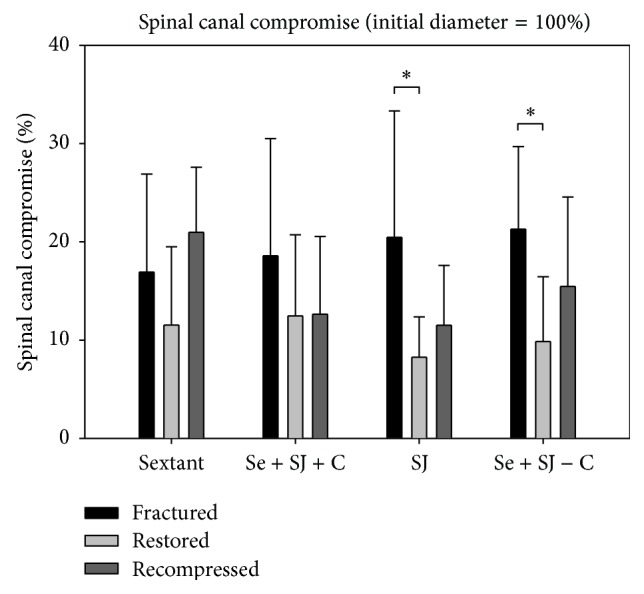
Bar graphs of the spinal canal compromise. The initial midsagittal diameter before fracture resembles 100%. Bars show the results for each of the four groups after fracture, after treatment, and after cyclic loading. Note that the use of an intravertebral reduction device led to clearance of the spinal canal. After cyclic loading the narrowing of the spinal canal increased in the dorsal instrumentation group.

Involvement of the posterior wall was meant to be a relative contraindication for the use of intravertebral reduction devices [19]. It has been shown that balloon kyphoplasty can be used in burst fractures in the elderly population [22]. The problem with using a balloon in burst fractures is the potential risk of pushing a posterior wall fragment further into the spinal canal. But, if the posterior ligaments are intact, the SpineJack can indirectly clear the spinal canal by optimising the ligamentotactic effect. When comparing the spinal canal compromise after fracture and after treatment it was cleared by all procedures (Figure 8).

**Figure 9:**
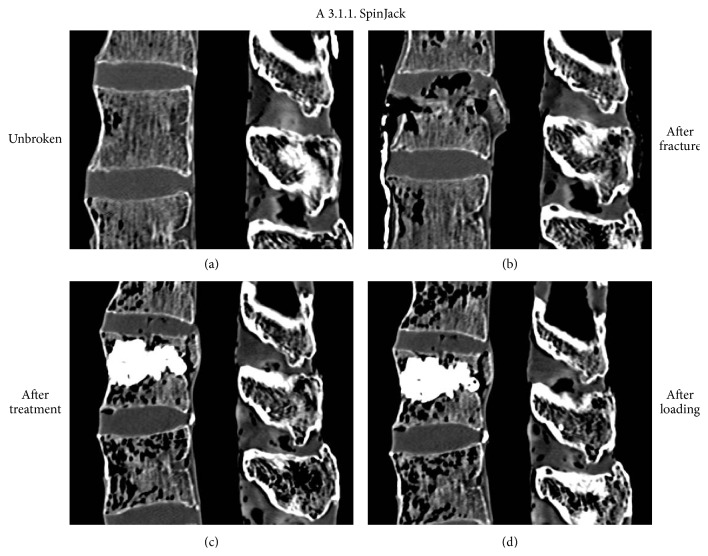
Representative case of an incomplete cranial burst fracture A 3.1.1. treated with intravertebral reduction device and cement augmentation. CT scans in the midsagittal plane before (a) and after (b) fracture, as well as after treatment (c) and after cyclic loading (d). Note the reduction of the endplate and clearance of the spinal canal. Both could be maintained after cyclic loading.

**Figure 10:**
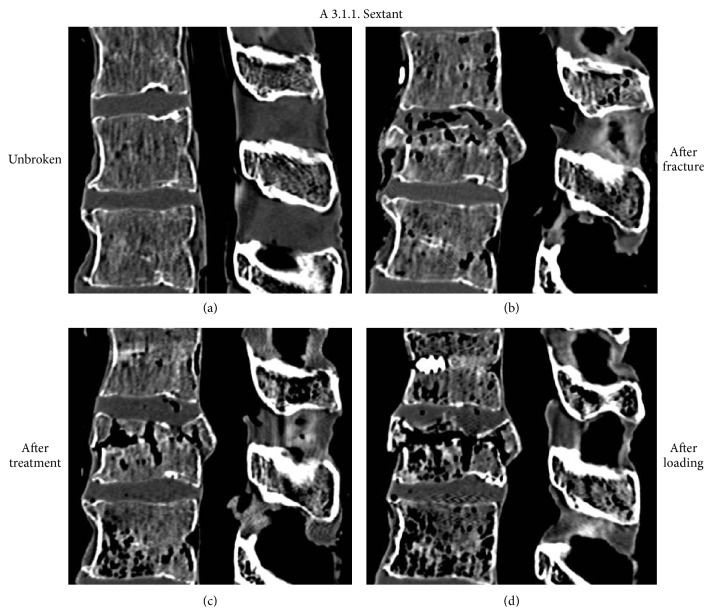
Representative case of an incomplete cranial burst fracture A 3.1.1. treated with sole dorsal instrumentation. CT scans in the midsagittal plane before (a) and after (b) fracture, as well as after treatment (c) and after cyclic loading (d). Note that after treatment limited height restoration was achieved. This could not be maintained after cyclic loading.

Although there was a large standard deviation, the results were significant for the groups PI + SJ + CE and PI + SJ without cement (Figures 8 and 9). After loading, the clearance of the spinal canal was better maintained in all SJ groups but even then the results were not significant (*p* > 0.05). By contrast, the spinal canal compromise was greater after loading when the stand-alone dorsal instrumentation was used (Figure 10). These values might be another strong argument for the additional value of using SJ together with dorsal instrumentation.

Burst fractures with involvement of the endplates have to be carefully evaluated before surgery. The SpineJack, like any other surgical device, bares the potential risk of being misplaced by the surgeon. In this experiment penetration of the wings of the device through the endplates was not observed.

*Limitations of the Study. * The study was performed on human cadaveric spines at 37°C to simulate the cementing technique as physiologic as possible. Like any other biomechanical study the results have to be critically interpreted before transferring techniques in the clinical setting. With heterogeneity of the specimen and a defined trauma mechanism fractures became comparable.

The specimens were not fixed in a loading apparatus during CT scans; this might also have influenced the results. This study was an attempt to understand the value of intervertebral reduction devices better. Future biomechanical and clinical studies are needed.

## 6. Conclusion

Based on the results of this biomechanical cadaver study, the SpineJack has shown its capabilities as a reduction device in the treatment of traumatic burst fractures. With regard to height restoration and, more importantly, anatomical restoration, the combination of the SpineJack with dorsal instrumentation showed significantly better results than the well accepted standard of sole dorsal instrumentation in particular.

Clinical implications include better restoration and maintenance of the sagittal balance of the spine and a reduction of deformity, especially of the endplates and posterior wall fragments, and this may relate to the clinical outcome and the biological healing process.

Additional studies with different cement volumes, fillings, and types will help us understand the situation better.

## Figures and Tables

**Figure 7:**
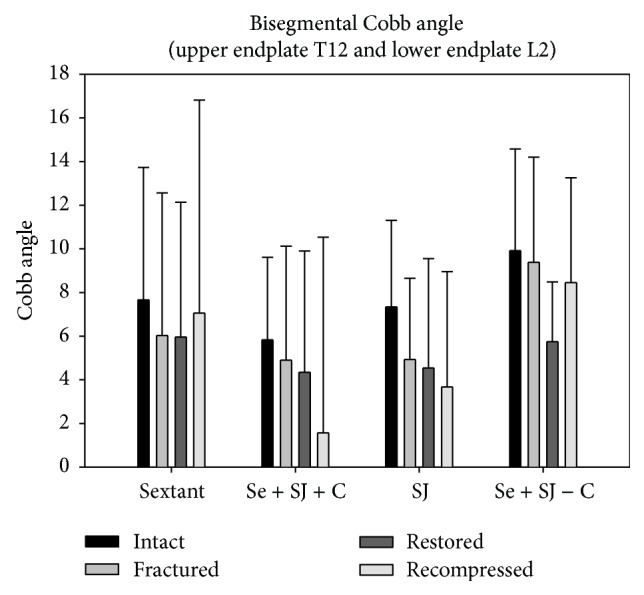
Measurements of the bisegmental Cobb angle. Bars show the results for each of the four groups before and after fracture, as well as after treatment and after cyclic loading.

**Table 1:** Overview of the results. All values are expressed as percentages of the initial nonfractured vertebral height. The bisegmental Cobb angle was measured between the upper endplate of T12 and the lower endplate of L2 in degrees. Group 1 = posterior instrumentation only (PI); Group 2 = PI + SpineJack + cement (PI + SJ + CE); Group 3: SpineJack + cement (SJ + CE); Group 4: SI + SpineJack (PI + SJ). Statistical significant differences (Tukey's Test *p* < 0.05) are marked with an asterisk^*∗*^.

Initial unfractured values equal 100%	PI	PI	PI + SJ + CE	PI + SJ + CE	SJ + CE	SJ + CE	PI + SJ	PI + SJ
	Mean	STDV	Mean	STDV	Mean	STDV	Mean	STDV
Fractured anterior height (%)	88.1	7.2	87.7	9.9	86.9	9.1	90.2	4.5
Fractured central height (%)	71.7	8.4	72.7	12.4	71.1	13.0	72.9	11.8
Fractured posterior height (%)	85.7	5.3	85.2	8.8	87.0	3.3	89.5	6.1
Restored anterior height (%)	94.2	6.3	102.1	7.9	102.8	6.6	100.8	4.1
Restored central height (%)	69.4	13.8	98.4^*∗*^	6.0	99.1^*∗*^	7.7	98.9^*∗*^	12.8
Restored posterior height (%)	85.9	5.8	95.9^*∗*^	4.0	100.4^*∗*^	6.5	94.8^*∗*^	5.1
After cyclic loading anterior height (%)	85.2	7.8	99.7^*∗*^	5.7	101.0^*∗*^	5.9	95.8^*∗*^	4.4
After cyclic loading central height (%)	63.7	12.2	95.7^*∗*^	5.8	96.7^*∗*^	6.6	85.0^*∗*^	9.8
After cyclic loading posterior height (%)	80.9	6.7	96.1^*∗*^	3.2	96.7^*∗*^	5.0	89.4^*∗*^	7.1
Narrowing of the spinal canal (after fracture; %)	16.9	10.0	18.6	11.9	20.5	12.9	21.3	8.4
Narrowing of the spinal canal (after restoration; %)	11.5	8.0	12.5	8.3	8.3	4.1	9.9	6.6
Narrowing of the spinal canal (after loading; %)	21.0	6.6	12.5	7.9	11.5	6.1	15.5	9.1

Cobb angle after fracture	6.0	6.5	4.9	5.2	4.9	3.7	9.4	4.8
Cobb angle after restoration	6.0	6.2	4.3	5.6	4.5	5.0	5.7	5.7
Cobb angle after cyclic loading	7.1	9.8	1.6	9.0	3.7	5.3	8.5	4.8
